# A Novel Side-Chain Orientation Dependent Potential Derived from Random-Walk Reference State for Protein Fold Selection and Structure Prediction

**DOI:** 10.1371/journal.pone.0015386

**Published:** 2010-10-27

**Authors:** Jian Zhang, Yang Zhang

**Affiliations:** Center for Computational Medicine and Bioinformatics, University of Michigan, Ann Arbor, Michigan, United States of America; Leeds Institute of Molecular Medicine, United Kingdom

## Abstract

**Background:**

An accurate potential function is essential to attack protein folding and structure prediction problems. The key to developing efficient knowledge-based potential functions is to design reference states that can appropriately counteract generic interactions. The reference states of many knowledge-based distance-dependent atomic potential functions were derived from non-interacting particles such as ideal gas, however, which ignored the inherent sequence connectivity and entropic elasticity of proteins.

**Methodology:**

We developed a new pair-wise distance-dependent, atomic statistical potential function (RW), using an ideal random-walk chain as reference state, which was optimized on CASP models and then benchmarked on nine structural decoy sets. Second, we incorporated a new side-chain orientation-dependent energy term into RW (RWplus) and found that the side-chain packing orientation specificity can further improve the decoy recognition ability of the statistical potential.

**Significance:**

RW and RWplus demonstrate a significantly better ability than the best performing pair-wise distance-dependent atomic potential functions in both native and near-native model selections. It has higher energy-RMSD and energy-TM-score correlations compared with other potentials of the same type in real-life structure assembly decoys. When benchmarked with a comprehensive list of publicly available potentials, RW and RWplus shows comparable performance to the state-of-the-art scoring functions, including those combining terms from multiple resources. These data demonstrate the usefulness of random-walk chain as reference states which correctly account for sequence connectivity and entropic elasticity of proteins. It shows potential usefulness in structure recognition and protein folding simulations. The RW and RWplus potentials, as well as the newly generated I-TASSER decoys, are freely available in http://zhanglab.ccmb.med.umich.edu/RW.

## Introduction

The basic hypothesis of protein folding theory is that protein structure generally has the lowest Gibbs free energy in the native state [1]. Therefore, an accurate energy function is the key to solve the protein folding and protein structure prediction problems. The commonly used potential function can be divided into two categories [2]. The first is physics based potential [e.g. AMBER [3], CHARMM [4] and GROMOS [5] etc], which can in principle be derived from the laws of physics. Although atomic-level structure refinement can be achieved with the molecular dynamics (MD) simulations in some isolated instances, no systematic structure improvement has been observed [6], [7], [8]. The second is knowledge-based potential [e.g. RAPDF [9], KBP [10], DFIRE [11], DOPE [12], OPUS-PSP [13], [14], free-rotating chain-based potential [15], or the more composite TASSER/I-TASSER [16], [17], [18] and ROSETTA [19] potentials], which is derived from the statistical regularities [20] of the solved protein structures in the PDB library [21].

The Knowledge-based potentials include contact potentials [22], [23], orientation-dependent potentials [13], [14], [24], and distance-dependent potentials [9], [10], [11], [12], [25], [26], [27], [28]. According to the reference state calculations, the distance-dependent potentials can be further divided into two classes: that using statistical reference states [RAPDF [9] and KBP [10]] and that using analytical reference states [DFIRE [11] and DOPE [12]]. It has been argued that the analytical reference state potential has better performance [11], [12]. For example, the DFIRE potential used a reference state derived from a set of uniformly distributed non-interacting points in finite spheres [11]. DOPE [12] later introduced an improved reference state which used non-interacting atoms in a homogeneous sphere with the radius dependent on a sample native structure [12]. Both DOPE and DFIRE were derived from a non-interacting ideal gas reference state and the major difference is that DOPE also takes into account the size effect of proteins.

The Knowledge-based potentials were successfully applied to many areas, including fold recognition [23], [29], [30], [31], *ab initio* protein structure prediction [16], [19], [32], [33], [34], protein structure refinement [13], [14], [35], structural model assessment [9], [10], [11], [12], protein-protein docking [22] and protein stability prediction [11], [22]. Despite the success of the potentials, more accurate accounting of atomic interactions will undoubtedly increase the power of the potentials in each of the application areas. In general, a protein is essentially a continuous sequential chain of the amino acid residues. The reference state, which accounts for the expected number of atom pairs at certain distance when interactions vanish, should correctly reflect and counteract the inherent chain connectivity effect. This feature, however, cannot be captured by the current ideal gas based reference state. Recently, Cheng *et al.* showed that a more physical reference model, such as free-rotating chain-based potential, could improve the performance of statistical potentials [15]. Aloy and Oliva introduced a method to split the knowledge-based potentials in biologically meaningful terms which allows a better combination of most relevant scoring functions [36]. Rykunov and Fiser performed a systematic comparison of publicly available scoring functions on CASP decoys which shows a critical role of reference state definitions. Based on the observation, the authors developed a residue based potential that employs a shuffled reference state with considering side-chain orientations and demonstrates advantages in structure decoy recognition [37].

In this work, we proposed a new distance-dependent atomic potential using a random-walk ideal chain as the reference state. This reference state was derived from a linear freely-jointed chain model, which can be considered as the segments of an ideal polymer chain performing a random walk (or “random flight”) in three dimension space. We term the new potential “RW potential”. The orientation-dependent all-atom potential, such as OPUS-PSP (it used a set of 19 rigid-body blocks extracted from the chemical structures of all 20 amino acid residues), can capture the feature of side-chain packing [13]. In this paper, a new orientation-dependent potential term was also added to RW. 20 vector pairs were defined to describe the side-chain orientation of 20 amino acids. The orientation term was then generated from the orientation specific packing statistics of those vector pairs in a non-redundant high-resolution structural database. The RW potential and the hybrid potential (RWplus) were optimized on CASP models and tested on eight commonly used decoy sets, as well as a new decoy set from real-life I-TASSER structure assembly followed by MD refinements. Detail comparisons with the state-of-the-art potentials demonstrated the advantage of the new reference state of chain connectivity and the side-chain orientation specificity.

## Results

We tested our potential in three ways: (1) the ability to select native structures from structural decoys; (2) the ability to select the best models from structural decoys when the native structures are excluded; (3) the correlation between the potential and the similarity (TM-score and RMSD) of the structural decoy to the native.

As a control, we compared the results of RW and RWplus mainly with two frequently used atomic potentials, DFIRE [11] and DOPE [12]. DFIRE was developed by Zhou and Zhou [11] and we calculated the DFIRE score by the DFIRE program, which is provided by the authors (http://sparks.informatics.iupui.edu/download/ddfire_bin.tgz) [38]. DOPE was developed by Shen and Sali [12] and we calculated DOPE scores from the MODELLER-9v7 package (http://salilab.org/modeller). In the end of the section, we presented a comparison of RW and RWplus with all potentials listed in the Rykunov and Fiser benchmark set [37].

### Testing on native structure selection

The ability of native structure selection of DFIRE, DOPE, RW and RWplus is tested using eight independent decoy sets (see Methods), where the experimental structures are mixed with other decoys generated by computers. The purpose is to rank the native structure as the lowest energy conformation using automatic scoring. Meanwhile, the significance of the energy of the native structures (*E*_native_) is evaluated by the normalized energy gap between *E*_native_ and the average energy of all decoys (*E*_aveage_), i.e. 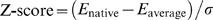, where σ is the energy deviation of all decoys.

The results of RW, RWplus, DFIRE and DOPE on the native structure selections are listed in Table 1. While there are some fluctuations for the selection ability of different potentials among different decoy sets, RWplus potential correctly identified 123 native structures for a total of 168 targets with a success rate of 73%. The RW potential correctly identified 120 native structures for a total of 168 targets with a success rate of 71%. DFIRE and DOPE were successful for 115 and 98 targets, resulting in a total success rate of 68% and 58% respectively. The improvement of RW and RWplus was also reflected by the Z-score of the native structures. The average Z-scores for all eight decoy sets were −4.03 for RWplus and −3.23 for RW, compared to −2.94 for DFIRE and −2.47 for DOPE. Among the eight decoy sets, RWplus and RW have the lowest Z-scores for six decoy sets (fisa, fisa_casp3, lmds, lattice_ssfit ROSETTA and I-TASSER). For the remaining two decoy sets (4state_reducred and Moulder), the Z-scores of all potentials are worse than −4.0 and the selections of different potentials are somewhat random. This is mainly due to the quality of the decoy sets, for example, having poorly packed native structures.

**Table 1:** Performance on native structure recognition.

Decoy sets	DFIRE	DOPE	RW	RWplus	#Targets
4state_reduced	6 (−3.44)	**7** (**−3.66**)	6 (−3.45)	6(−3.54)	7
Fisa	3 (−4.67)	3 (−3.91)	3 (−4.87)	3(**−4.96**)	4
fisa_casp3	3 (−4.93)	3 (−5.06)	**4** (**−5.22**)	**4**(−5.14)	5
Lmds	7 (−0.99)	7 (−1.34)	7 (−1.20)	7(**−4.28**)	10
lattice_ssfit	8 (−8.00)	8 (−7.43)	8 (−8.15)	8(**−8.59**)	8
Moulder	19 (−2.79)	19 (**−3.09**)	19 (−2.79)	19(−3.04)	20
ROSETTA	22 (−1.67)	21 (−1.61)	20 (−1.62)	20(**−2.30**)	58
I-TASSER	47 (−3.58)	30 (−2.18)	53 (−4.42)	**56**(**−5.38**)	56
#Total(Z-score)	115 (−2.94)	98 (−2.47)	120 (−3.23)	**123**(**−4.03**)	168

The data shows the number of targets which have the native structure ranked as the lowest energy. The values in parenthesis are the average Z-score of the corresponding potentials. The highlights are those having the highest number in each category.

RW with additional orientation energy term has a consistent better performance than RW. The average Z-scores of RWplus are lower than RW for seven out of eight decoy sets and the successful selection rate of RWplus is 2% higher than RW. This improvement is due to the contribution of the orientation dependent energy term, which cannot be counted by the pair-wise distance dependent potential. With orientation energy term, the most-probable side-chain packing patterns in high-resolution experimental structures, such as π-π and cation-π interactions, can be correctly recognized and be assigned lower orientation energy than the less favorite patterns. Thus the RWplus energies of the native structures are lower than RW and average Z-scores values of RWplus are much better.

### Selection of best models from I-TASSER and ROSETTA decoys

The ability to identify native structure from structural decoys is only a minimum request to measure the potentials. Although the selection of the native structures has been a common goal of many protein potential developments [9], [10], [11], [12], [39], the usefulness of the criterion is limited. First, there are no native structures which are generated from computer simulations, and all computer models of structure predictions have some level of errors. Second, since the experimental structures are usually perfect conformations in many aspect of features (i.e. H-bonding, atomic clashes, secondary structure regularities, rotamer optimizations, electrostatics interactions etc), it is a relatively easy task to pick out the native structure from a set of computer decoys. On some occasions, a simple counting of some special features (e.g. the atomic clashes) may be enough to distinguish the native structures from the roughly generated computer decoys. So, in what follows, we focus on the more challenging and realistic cases of identifying the best decoys from real-time simulations by I-TASSER [34] and ROSETTA [40], [41], or examining the correlation of the energy with the quality of decoys (i.e. RMSD and TM-score to the native). In this respect, we do not consider the decoy sets generated by manual variation of the native structures because the quality of the decoys usually has a strong correlation with the radius of gyrations. We also exclude the decoy sets from homologous modeling because the decoys are usually biased to specific templates and the distance to the initial template may be an efficient metric for decoy recognition [18].

We used RMSD and TM-score as the two criteria for assessing the quality of every structural decoy. RMSD is defined as the root mean squared derivation of all Cα pairs of the decoy to the native structure. Because RMSD weights all distances equally, it is insensitive to the global topology for large RMSD of decoys (e.g. a mis-oriented decoy may have a big RMSD although the global topology in the core region is correct). TM-score [42] weights the large distance at a small weight which makes the magnitude of TM-score more sensitive to the topology rather than the outlier of the structures [43], [44]. TM-score ranges in (0, 1] where higher values indicate better quality.

Table 2 summarizes the result of best model selection by DFIRE, DOPE, RW and RWplus for the TASSER decoys. If we consider the first model as ranked by the lowest energy, the average RMSD of the first models by RW is 5.20 Å which is 0.4 Å and 0.1 Å lower than that by DFIRE and DOPE, respectively. RWplus has the lowest average RMSD 5.19, which is slightly better than RW. The average TM-score of the first model selected by RW is 0.569 which is also higher than that obtained by DFIRE (0.558) and DOPE (0.560) and RWplus has the best average TM-score (0.575). Apparently, none of the methods could select the absolute best structure as the highest rank model in the decoy sets, which has an average RMSD/TM-score = 3.3 Å/0.675. We also consider the quality of the best decoys which are in the top-five and top-ten lowest-energy decoys in Table 2. The selected models by RW are consistently closer to the native structure than those by DFIRE and DOPE.

**Table 2:** Average RMSD (Å) and TM-score (in parenthesis) of models selected from I-TASSER and ROSETTA decoy sets.

		DFIRE	DOPE	RW	RWplus
I-TASSER Decoys	First model	5.61 (0.558)	5.31 (0.560)	5.22 (0.569)	**5.19** (**0.575**)
	Top-five	4.45 (0.612)	**4.21** (0.613)	4.30 (**0.616**)	4.29 (0.608)
	Top-ten	3.95 (0.632)	3.89 (0.631)	3.89 (**0.633**)	3.89 (0.625)
ROSETTA Decoys	First model	**7.36** (**0.469**)	7.43 (0.466)	7.62 (0.460)	7.48 (0.464)
	Top-five	6.08 (0.533)	6.10 (0.536)	6.04 (**0.537**)	**6.01** (0.525)
	Top-ten	5.79 (0.559)	5.85 (0.555)	5.78 (**0.560**)	**5.76** (5.42)

The highlights are the highest value in each category.

Despite of the advantage of RW compared with other methods, we found that it could not select models better than those selected by the structure clustering program SPICKER [45], which was designed to identify the most frequently occurred structural state in the simulation. When we cluster the 500 decoys of I-TASSER, where the redundant decoys have been removed, the average RMSD and TM-score for the first model (CLOSC) are 4.99 Å and 0.572, respectively. If we run SPICKER in the original I-TASSER trajectories (i.e. the 12,500–32,000 conformations which include structural redundancy), the RMSD and TM-score for the first model are 4.84 Å and 0.589, respectively. Here, CLOSC in SPICKER is the structure decoy which is the closest to the cluster centroid (COMBO) where the COMBO structure is calculated by averaging all structural decoys in the cluster. Because the cluster identified by SPICKER has the highest multiplicity and partition function 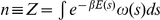 where s is the conformation phase space and ω is state density, it is actually selecting the state of the lowest Helmholtz free-energy, i.e. 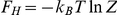. These results show the advantage of selecting models from the lowest of inherent free-energies.

In Table 2, we also present the result of near-native structure selections by DFIRE, DOPE, RW and RWplus for the ROSETTA decoys. RW potential consistently selected models closer to the native structure than those by DFIRE and DOPE in the top-five and top-ten lowest-energy decoys, while DFIRE selected the best first models with an average TM-score 0.469, which is slightly better than DOPE (0.466) and RW (0.460).

### Correlation between potential score and modeling errors

Except for the ability of selecting good models from structure decoys, another important criterion of assessing the potential development is to examine the correlation of the potential with the similarity of decoys to the native structure [16]. This is to some extent more important to protein folding because it can determine how structure assembly simulations are guided to the near-native states. Certainly, a golf-hole-like potential may be perfect in selecting good models but it is useless in protein folding because it lacks a middle-range funnel in such an energy landscape.

**Figure 1:**
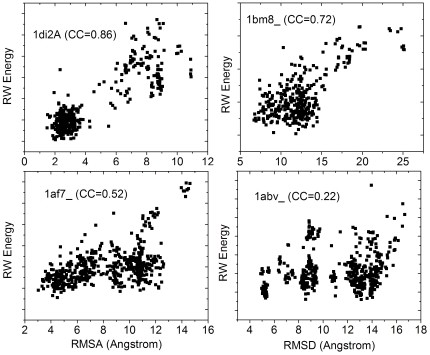
Illustrative examples of the correlations between the RW potential and the RMSD to native of the I-TASSER decoys. The number shows the Pearson correlation coefficients.

In Table 3, we present the Pearson correlation coefficients between Cα RMSD (and TM-score) and the potential energies as given by DFIRE, DOPE RW and RWplus for the I-TASSER decoys. Overall, RWplus has the best correlation coefficients. RW and DFIRE have comparable correlation coefficients although the average correlation coefficient of RW is slightly higher than that of DFIRE. The correlation coefficients of all three potentials are much higher than DOPE. More specifically, the RWplus potential yields an average energy-RMSD correlation coefficient of 0.53, compared with that of RW (0.52), DFIRE (0.51) and DOPE (0.32). The average energy-TM-score correlation coefficients are −0.52 for RWplus, −0.50 for RW, −0.49 for DFIRE, and −0.32 for DOPE. Four typical examples from 1di2A, 1bm8_, 1af7_ and 1abv_, which span different levels (strong/medium/weak) of RW-RMSD correlations, are shown in Figure. 1. A complete set of correlation plots are available at http://zhanglab.ccmb.med.umich.edu/decoys.

**Table 3:** The Pearson correlation coefficients between energy and Cα RMSD (CC-RMSD) and TM-score (CC-TMscore) for the I-TASSER and ROSETTA decoys.

	CC-RMSD				CC-TMscore			
Decoys	DFIRE	DOPE	RW	RWplus	DFIRE	DOPE	RW	RWplus
I-TASSER	0.514	0.319	0.520	**0.528**	−0.492	−0.317	−0.500	**−0.517**
ROSETTA	0.440	0.421	**0.441**	0.435	−0.432	−0.427	**−0.434**	0.427

The highlights are the highest value in each category.

The average energy-RMSD and energy-TM-score correlation coefficients for the ROSETTA decoys are also listed in Table 3. Again, RW and DFIRE have comparable correlation coefficients with the correlation of RW being slightly higher, while both of these are obviously higher than DOPE. RWplus has correlation coefficient between RW and DOPE for the ROSETTA decoys.

### Comparison with other potentials in the Rykunov and Fiser benchmark set

A comprehensive benchmarking survey of quality assessment scoring functions relative to a list of other publicly available potentials is shown in Table 4. The data of the potentials were adopted from Rykunov and Fiser [37] who compared the GDT_TS scores of the models recognized by each of the potentials. The model decoys for the 143 protein targets were generated during CASP5-CASP8 experiments. Data in Table 4 are sorted by the average rank of the lowest energy decoy structure according to the GDT_TS score for the decoy set excluding the native structure. To obtain the correct GDT_TS scores and RW and RWplus scores, the models in Rykunov and Fiser decoy set were first cleaned up by removing the remarks and hetero atoms and the residue numbers in the models were then reordered according to the native structures. The data of RWplus and RW were calculated from the cleaned Rykunov and Fiser's decoy set, which can be downloaded from http://zhanglab.ccmb.med.umich.edu/RW/casp_good.tar.gz. The RWplus and RW ranked second and third place respectively and have comparative performance to the best potential QMEAN6 [46] for average rank with and without native structures, which is a composite potential combining six structural descriptors including distance, solvation, torsion, secondary structure predictions [46].

**Table 4:** Performance of various potentials on selecting models generated in CASP5-8 experiments as collected by Rykunov and Fiser [37].

Scoring function	models only		native included	
	Average^a^	ranked 1^b^	Average^c^	ranked 1^d^
QMEAN6	2.87	85	1.71	113
**RWplus**	**2.97**	**57**	**1.78**	**106**
**RW**	**3.08**	**51**	**1.71**	**110**
QMEANall_atom	3.59	74	1.71	119
QMEANSSE_agree	3.74	62	3.72	39
QMEANACC_agree	4.04	40	3.78	48
RF_CB_SRS_OD	4.16	61	2.08	110
RF_CB_OD	4.62	62	2	111
RF_HA_SRS	4.65	49	1.38	137
RF_CB_SRS	4.72	56	2.18	114
OPUS_CA	4.72	79	5.13	55
VSCOREcombined	4.79	53	2.2	117
QMEAN-pairwise	4.8	54	3.15	85
Rosetta	5.01	57	4.09	68
Dong-pair	5.01	58	6.32	4
RF_CB	5.06	52	2.46	106
VSCORE-pair	5.08	54	1.85	128
PROSAcombined	5.11	57	3.38	87
OPUS_PSP	5.39	54	2.99	118
RF_HA	5.44	62	2.78	112
DOPE	5.77	54	3.27	95
dFIRE	6.03	50	5.69	33
PROSA-pair	6.03	56	3.54	95
QMEAN-torsion	6.71	45	3.24	114
Shortle2006	6.85	35	1.79	129
Liang_geometric	6.88	44	2.48	114
QMEANsolvation	7.32	33	6.27	54
Shortle2005	7.73	42	3.39	109
Floudas-CM	7.75	38	7.05	42
Floudas-Ca	7.79	33	8.36	10
NAMD_1000	8.06	24	4.96	78
Melo-ANOLEA	9.62	19	5.19	86
PC2CA	9.75	19	5.06	85
Melo-NL	9.99	14	5.85	80
NAMD_1	11.91	5	10.98	24
Random^e^	9.72	13.9	10.1	8.3

a The average rank of lowest energy decoy according to GDT_TS score (over 143 decoy sets) in the absence of native structures.

b The number of sets when the best model was ranked as first, in the absence of native structures.

c The average rank of the lowest energy decoy in GDT_TS when native structures are present.

d The number of sets when the best model was ranked as first when native structures are present.

e Expected random values were generated by picking a wining model fromthe decoy sets randomly. Average values over 1000 random trials are shown [37].

The performance of RWplus and RW varies depending on the presence or absence of the native structures. RWplus outperforms RW for average rank without native structures, but has worse performance for the average rank with native structures. RWplus can correctly select 57 best decoys for models without native structures which is 6 more than RW, whereas RW can correctly select 110 best decoys which is 4 more than RWplus. RW has significant better performance than other pair-wise distance dependent potentials of the same type of statistics, such as DFIRE [11] and DOPE [12], which indicate that the RW reference state, which mimics the entropic elasticity and chain connectivity, are efficient to counteract generic interactions.

## Discussion

### Comparison of different pair-wise distance dependent statistical potentials

**Figure 2:**
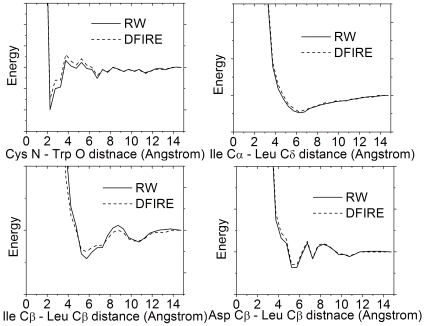
Distance dependence of DFIRE and RW potentials for four representative atom pairs.

Most of the atomic statistical potentials in the literature used the same equation with the major difference in the derivation of the reference state. To examine the detailed differences of the overall potentials, we compared in Figure 2 the distance dependence of RW and DFIRE potentials for four representative pairs of atom types in main chain–main chain, main chain–side chain, hydrophobic side chain–hydrophobic side chain, and polar side chain–hydrophobic side chain groups.

For all four pairs, RW potential has a steeper repulsion at short distance than DFIRE, and thus can assign a lower energy to the atom pairs with a favorite distance and give favorable structure lower energy. For example, the Ile C-beta atom–Leu C-beta atom pair has a deeper valley at 6Å and a higher peak at 9Å, which increases the energy gap between good pairs and bad pairs and therefore also increases the sensitivity of the potential to the structural variations. These subtle changes are mainly due to the difference in calculating the reference state where DFIRE considers the reference state as idea gas and RW treats it as a freely-joint chain with chain connectivity. The overall distance dependences of the potentials are qualitatively similar, because similar statistics were taken from the high-resolution experimental structures in the PDB library.

### Comparison of reference states of different pair-wise distance dependent potentials

To examine directly the reference states of DFIRE, DOPE and RW potentials, we calculated the ratio of reference state at a distance R to that at a distance cutoff 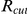 ( = 15Å) for a protein of 100 amino acids. For DFIRE, the expected number of atom pairs (α, β) in the distance shell 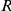 to 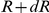 [11] is
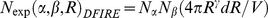
(1)where *V* is the volume of the ideal gas system and γ = 1.61. *N_α_* and *N_β_* are the number of atoms of type α and β, respectively.

For DOPE, the potential is derived from the distance probability density function [12]:
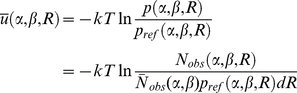
(2)where, 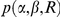 and 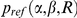 are the observed and reference distance probability density function of atom pair (α, β), respectively. 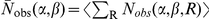 is the average number of observed atom pairs (α, β). Since 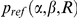 equals to the normalization function 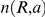 [12] and
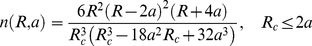
(3)where 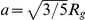 is the size of the sample protein structure and 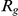 is the radius of gyration, the expected number of atom pairs (α, β) in the same distance shell can be written as
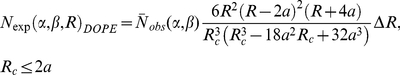
(4)where 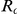 is some upper bound of the statistical potential.

**Figure 3:**
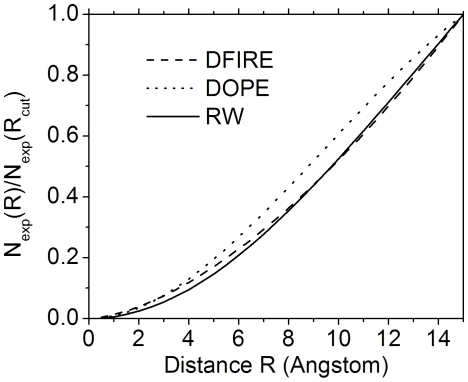
The ratio of reference state at a distance *R* to that at 15 Å versus *R* for FIRE, DOPE and RW potentials for a protein of 100 AA.

From Eq. (18), we can obtain the expected number of atom pairs (α, β) in the same distance shell for RW
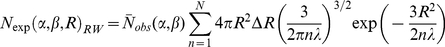
(5)In Figure 3, we present the ratio of reference states at distance R to that at 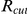 versus R for FIRE, DOPE and RW. It is shown that the RW potential has a lower ratio than DFIRE and DOPE at short distance, whereas at long distance the ratio of RW is similar to that of DFIRE but lower than that of DOPE. This difference makes the RW potential a steeper potential at short distance as showed in Figure 3 and therefore help increase the sensitivity of the potential to the short range interactions.

### Estimation of Kuhn length b and distance cutoff R_0_

There are two tuning parameters, the Kuhn length b and distance cutoff R_0_, in the RW potential derivation.

The Kuhn length b was introduced to match the scale of the FJC with that of real protein chains. We found that the optimized value of λ( = b2), at which RW achieves the best performance, equals to 460; this corresponds to a Kuhn length b = 21.4 Å. The value coincides with the data of the single molecule stretching experiments with atomic force microscope [47] and laser tweezers [48], where the persistent length of the muscle protein titin is between 4 Å and 20 Å [47], [48], [49], which corresponds to 8–40 Å in the Kuhn length according to the polymer theory [50].

R_0_ is the distance cutoff where the atomic pair-wise interaction vanishes. Increasing the cutoff can in principle extract more information from protein structures and improve the accuracy. But the long distance signal may be unstable which therefore, may not be well matched by an analytical equation. By trial and error, we set R_0_ = 15.5 Å as the distance cutoff in RW, which is slightly larger than 15 Å used with DFIRE and DOPE.

### Conclusion

We have constructed a new transferable distance-dependent, atomic statistical potential RW, using an ideal random-walk chain of a rigid step length as the reference state. Because the ideal chain has no amino acid-specific interactions between the subunits but keeps the sequence continuity, it mimics the generic entropic elasticity and connectivity of polymer protein molecules, which could not be described by other reference states such as ideal gas systems used in DFIRE and DOPE. As a result, the RW potential has a steeper energy at short distances than these analytical potentials, which helps the RW potential to capture strong signals at short-range interactions. This is particularly important since the atomic potential in our modeling is essentially a short-range one. We also combined RW with a side-chain orientation-dependent energy term and built a hybrid potential RWplus. It is found that the orientation energy term does improve the ability of RW in recognizing the native-like structural features.

RW and RWplus have been extensively tested on nine sets of structural decoys from manual assembly, threading, homologous modeling, and *ab initio* simulations. RWplus correctly recognized the native structures in 73% of cases which is 5–15% higher than other state of the art pair-wise statistical methods. The RW potential selected better quality models than other distant-dependent statistical potentials from ROSETTA and I-TASSER simulations. When compared with a comprehensive list of publicly available other potentials, including composite potentials combining terms from multiple resources, RWplus and RW show comparable performance to the currently best quality assessment scoring functions for the decoy selections. The general correlation coefficient between the RW/RWplus potentials and the RMSD/TM-score is 0.50–0.53 for the I-TASSER decoys which is higher than DFIRE, and significantly higher than DOPE – although the correlation coefficients for the ROSETTA decoys are slightly lower for all potentials. This strong correlation, together with and the decoy recognition power, demonstrates the exciting probability of using the potential in improving the efficiency of protein folding and protein structure refinement algorithms. The corresponding work of employing RW and RWplus to I-TASSER based *ab initio* protein folding is in progress.

## Materials and Methods

### Construction of pair-wise distance dependent potential

A variety of distance-dependent, pair-wise, statistical potentials [9], [10], [11], [12], [20] are derived from the inverse of Boltzmann's law:
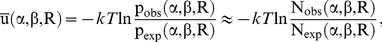
(6)where k is the Boltzmann constant and T is the Kelvin temperature; R is the distance between atoms of atom type α and β; 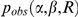 and 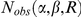 are the observed probability and number of atom pairs (α, β) within a distance shell R to 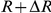 respectively; and 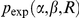 and 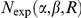 are the expected probability and number of atom pairs (α, β) in the same distance shell respectively when there is no interactions between atoms. The purpose of 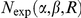 is to rule out by normalization the average and generic dependence of atom pairs (α, β) which do not stem from the atom-atom pair interactions. The method of counting 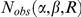 is the same among different methods while the method of calculating 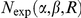 is what makes one potential differ from another. Because one of the major purposes of the potential 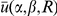 is to recognize the correct conformations from the structural decoys generated in the structural modeling simulations where the decoys are from continuous sequences in most cases, the generic feature of the chain connectivity is a major consideration for calculating 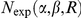 in our model.

Here, we applied the freely-jointed chain (FJC) model [50], [51] to construct a random-walk reference state, which keeps the general chain connectivity but has no long-range interactions between nodes except for the entropy elasticity that is generic in all protein structures. The expected number of atom pairs at a distance shell R for the FJC can be calculated by 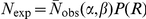, where P(R) is the probability for the atom pair in a spherical shell with radius between R and R+dR and where 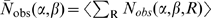 is the average number of atom pairs of type α and β in a protein molecule.

**Figure 4:**
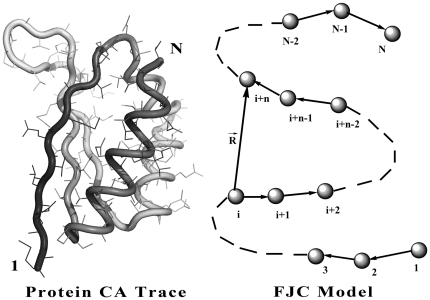
The illustration of random-walk ideal chain model and the relationship with real protein chain. A protein with *N* residues can be mapped to a freely-jointed chain with *N* subunits.

Consider a linear polymer to be a FJC with n subunits, each of the Kohn length b, which occupy zero volume so that no part of the chain excludes another, i.e. there is no interaction between the subunits (the excluded volume will be discussed later). One can regard the segments of each such chain in an ensemble as performing a random walk in the three-dimensional space. Since the atoms of distance R can be observed in the residue pairs of different order of distances along the chain, we first consider the conformation of FJC in a set of (n+1) position vectors 
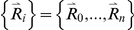
 of the joints, or alternatively, by the set of bond vectors 
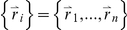
, where 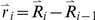 (Figure 4). Since the bond vectors 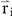 are independent of each other, the distribution function of the polymer conformation can be written as
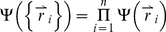
(7)where 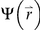 denotes the identical distribution of a vector of constant length b.

Let 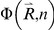 be the probability distribution function with the end-to-end vector of the chain consisting of n links of 
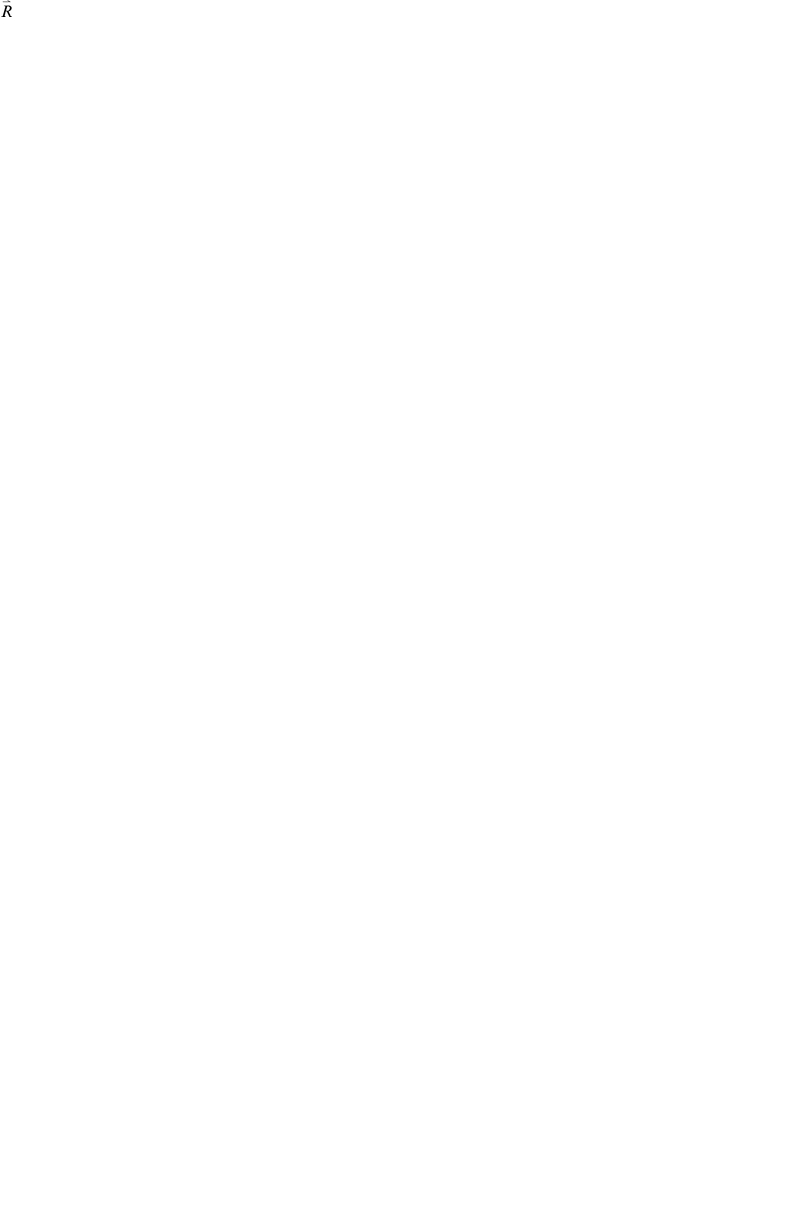
. Given the conformational distribution of 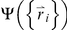, 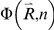 can be written as
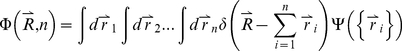
(8)where
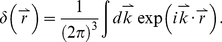
(9)Thus, we have
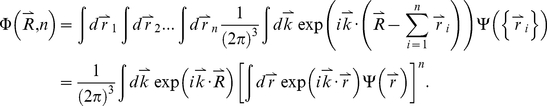
(10)Since 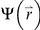 depends only on 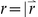, the integral 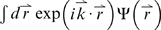 over the direction of 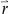 can be carried out as
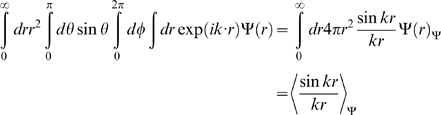
(11)where 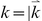. In the small k region, it can be approximated as
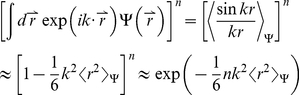
(12)For the FJC with a constant bond length b, we have 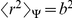, thus
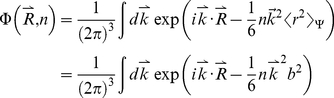
(13)Eq. (13) is a Gaussian function integration which can be explicitly carried out [52]. The probability distribution function can be written as
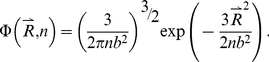
(14)As a function of the end-to-end distance 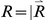, this probability distribution can be rewritten in the spherical coordinate system:
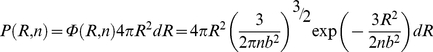
(15)The probability function of distance R for an atom pair with residue number i and i+n is the probability of the end-to-end vector 
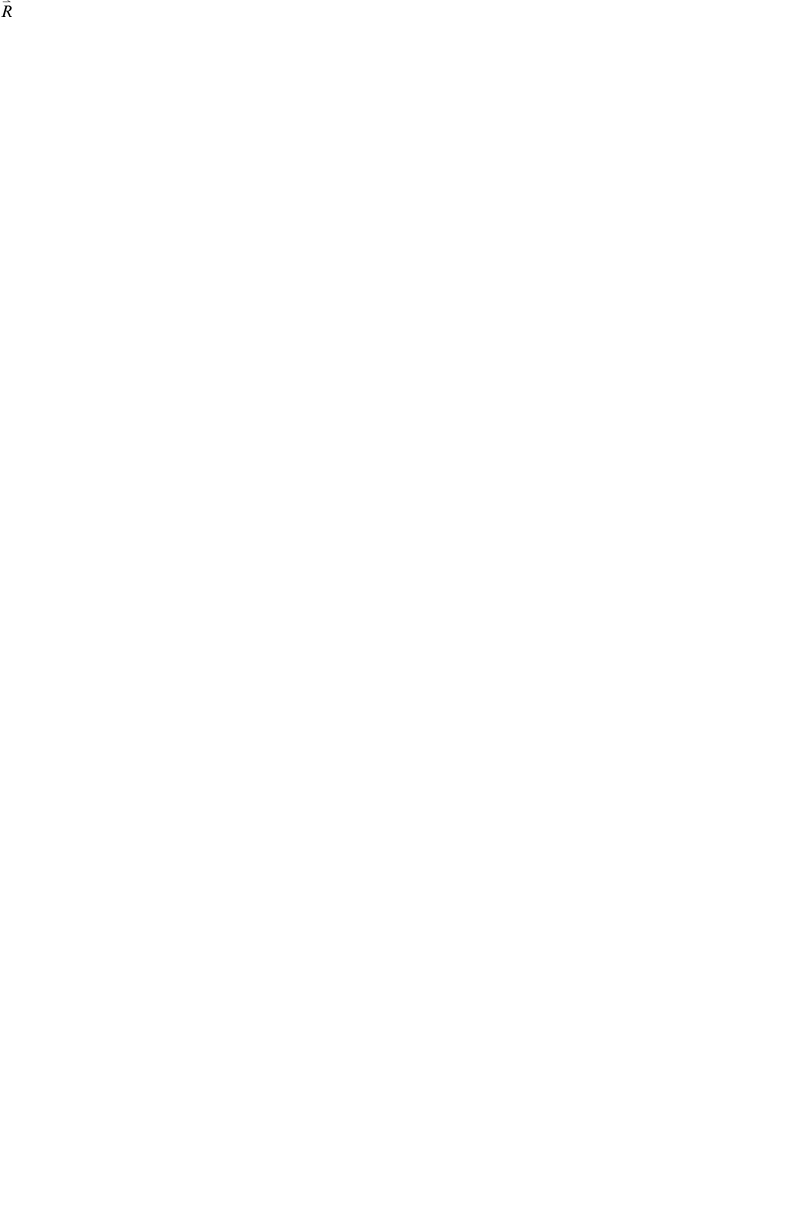
 being in the spherical shell with radius between R and R+dR if n is less than the protein sequence length N. In contrast, if n is larger than N, the probability function of distance R is zero, i.e.
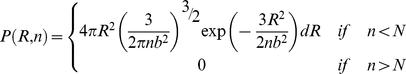
(16)Given all different order of residue distances, the probability of distance R is
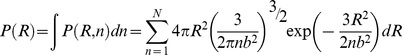
(17)Because the model developed here has mapped the FJC nodes to protein residues while the potential in Eq. (6) accounts for the interactions of protein atoms, there is no definite correspondence between the Kuhn length b of the FJC model and the residue scale of real proteins. Therefore, we consider 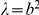 as a freely-tuned parameter to match the scale of the FJC with that of a real protein chain. The tuning of this parameter can also partially amend the generic excluded volume interactions of the protein chain which have not been considered in the derivation of the ideal FJC model. Thus, the final statistical potential equation is
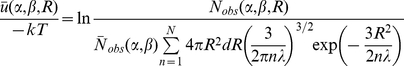
(18)Suppose 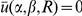 at certain distance R0, the potential can be rewritten as
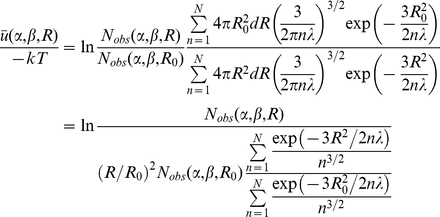
(19)where R_0_ is the second parameter tuned for identifying the location where the atomic pair-wise interaction vanishes.

### Construction of orientation dependent potential

**Figure 5:**
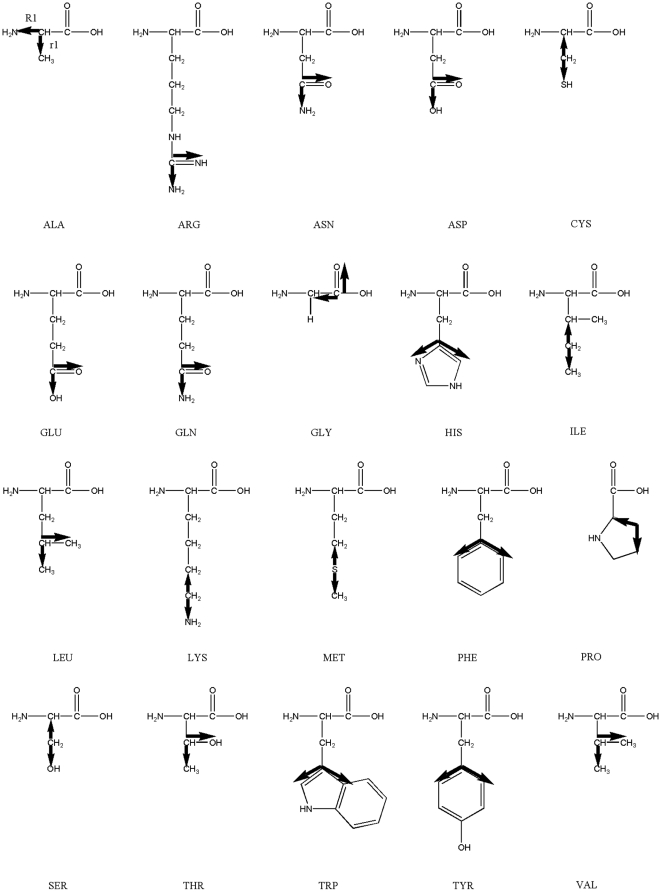
The definition of 20 vector pairs.

**Figure 6:**
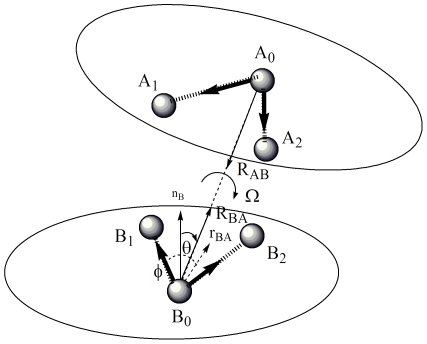
The definition of relative orientation of two vector pairs *A* and *B*. 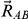
 is the direction vector from *A* to *B*. 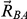 is the direction vector from *B* to *A*. Ω is the torsion angle between plane *A_1_AB* and plane *ABB_1_*.

To specify the side-chain packing orientation, we define 20 vector pairs as shown in Figure 5. For each residue type except GLY and ALA, a unique vector pair is defined based on three most representative side-chain atoms. Totally 18 vector pairs are used to represent the orientation of side-chain atoms and 2 vector pairs are used to represent the orientation of main-chain atoms. The relative orientation of two vector pairs (*A* and *B*) can be expressed by three variables: two direction vector 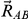 and 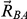 and a torsion angle 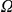 as shown in Figure 6. *A* is the vector pair of *A_0_A_1_* and *A_0_A_2_*, which represents the orientation of three side-chain atoms *A_0_*, *A_1_* and *A_2_*. *B* is the vector pair of *B_0_B_1_* and *B_0_B_2_*, which represents the orientation of three side-chain atoms *B_0_*, *B_1_* and *B_2_*. 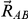 is the direction vector from *A_0_* to *B_0_*. 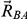 is the direction vector from *B_0_* to *A_0_*. 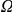 is the torsion angle of *A_1_A_0_B_0_B_1_*. We coarse-grained the orientation space into 2704 bins for two vector pairs due to the limited amount of available protein structure data and the balance between the number of bins and the available structure data for statistical analysis [13]. As illustrated in Figure 6, the direction vector 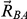 can be coarse-grained into 26 bins based on two parameters θ and ϕ, where θ and ϕ are the spherical angles of vector 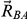 in the reference frame of *B_0_B_1_B_2_*. The definition of 26 bins is illustrated in Table 5. The direction vector 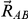 can also be coarse-grained into 26 bins in the same way. The torsion angle 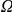 is coarse-grained into four bins spanning π/2 radians each. Thus, for two vector pairs, the number of bins is 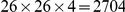.

**Table 5:** The definition of the relative direction bins for a direction vector and the probability of the reference state for each bin.

θΦ	(0,π/6)	(π/6,π/3)	(π/3,π2/3)	(π2/3,π5/6)	(π5/6,π)
(π/8,π3/8)	A	B	C	B	A
(π3/8,π5/8)		B	C	B	
(π5/8,π7/8)		B	C	B	
(π7/8,π9/8)		B	C	B	
(π9/8,π11/8)		B	C	B	
(π11/8,π13/8)		B	C	B	
(π13/8,π15/8)		B	C	B	
(π15/8,π3/8)		B	C	B	

A = 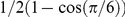.

B = 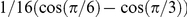.

C = 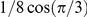.

To calculate the total orientation-dependent packing energy, we define the packing energy for two vector pairs *A* and *B* in relative orientation space using a similar Boltzmann formula as Eq. (6):
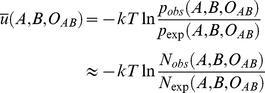
(20)Here, *OAB* is the relative orientation between vector types *A* and *B*; 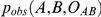 and 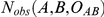 are the observed probability and number of vector pair (*A*, *B*) within a relative orientation *OAB* respectively and 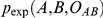 and 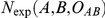 are the expected probability and number of vector pair (*A*, *B*) in the same relative orientation bin when there is no interactions between atoms.

If we assume that every two vector pairs have the same random orientation distribution for the reference state, we can calculate the expected number of vector pair (*A*, *B*) as:
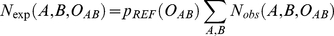
(21)where 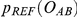 is the expected probability of relative orientation *OAB* in the reference state. We assume that every two vector pairs have no interactions in the reference state and the three orientation variables (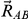, 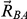 and 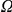) are independent. They should have random distributions in orientation space and 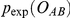 can be calculated as:
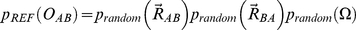
(22)where 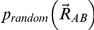 and 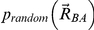 are the probability of a vector with random orientation in space and 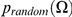 is the probability of a random torsion angle in four bins spanning π/2 radians each and should be equal to 0.25. 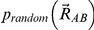 and 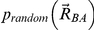 can be obtained by calculating the fraction of surface area for each bin in a spherical surface. The probabilities of 26 bins are listed in Table 5

### Construction of hybrid distance and orientation dependent potential

The hybrid potential E_RWplus_ is composed of a distance dependent energy term E_RW_ and an orientation dependent term E_orient_. Therefore the total energy can be calculated by the sum of energies of all distance pairs and vector pairs of non-consecutive residues:
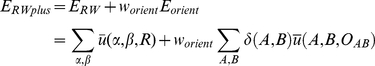
(23)Here, 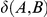 is 1 when vector pairs *A* and *B* are in contact (at least there is one heavy atom pair with distance less than 10 Angstrom) and 0 otherwise; w_orient_ is a weight parameter optimized against training decoy sets.

### Experimental structure database for potential statistics

1,383 high-resolution experimental structures were used to calculate the statistical potential. The non-redundant protein list was constructed with the PISCES web server [53], with a percentage identity cutoff 20%, a resolution cutoff 1.6 Å, and a R-factor cutoff 0.25 Å. For the RW potential calculation, the distance cutoff is 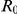. The pair distance from 0 to 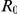 was divided into bins with a bin width dR = 0.5 Å. A total of 158 residue-specific atom types, same as DOPE [12], were used.

### Parameter training

The RW potential is trained on the conformations generated in the CASP7 and CASP8 experiments [54], [55]. This training set includes 203 single-domain targets taken from http://predictioncenter.org/download_area/CASP8 and http://predictioncenter.org/download_area/ CASP7. Only the decoys with full-length structures were considered and those with missed residues were removed for the convenience of potential evaluations. The final decoy set has 300 to 500 models for each target.

The RW and RWplus potential was optimized using the CASP decoys by tuning parameters λ and 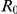 in Eq. (19) and w_orient_ in Eq. (23). The objective is to maximize the number of correctly selected native structures from decoys and the Pearson's correlation coefficient between the RW potential and the TM-score of the modeling decoys. When λ equals to 460, 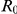 equals to 15.5 Å and w_orient_ equals to 0.1, we found that the potential has the best performance with an average Pearson's correlation coefficient with TM-score to the native structure of 0.64; due to the difficulty of the CASP decoy set, the native structure was correctly selected in only 77 out of 203 targets.

### Testing structural decoy sets

Eight multiple decoy sets, including the 4-state_reduced [56], fisa [57], fisa_casp3 [57], lmds, lattice_ssfit [58], moulder [59], ROSETTA [40] and I-TASSER decoys sets, were used to evaluate the performance of the statistical potential. The first five decoy sets are available through Decoys ‘R’ Us [60] (http://dd.compbio.washington.edu/).

The moulder decoy set by John and Sali is derived by iterative target-template alignment and comparative modeling of 20 target sequences that are remotely related to their template structures [59]; it contains 300 decoy models for each target, based on a wide range of target-template alignment accuracy (http://salilab.org/decoys).

The ROSETTA decoy set by Baker and coworkers [40], [41] contains 20 random models and 100 lowest scoring models from 10,000 decoys, which were generated for 58 small proteins using ROSETTA de novo structure predictions followed by all-atom refinement (http://depts.washington.edu/bakerpg).

The I-TASSER decoy set includes the atomic structure decoys generated for 56 non-homologous small proteins. The backbone structures were first generated by the I-TASSER ab initio modeling by Wu et al. [34], where for each protein target 12,500–32,000 conformations were taken from the trajectories of 3 lowest-temperature replicas of the simulations. Because this raw decoy set may contain redundant structures and some conformations have steric clashes, we select 300–500 non-redundant decoys for each target by iterative structure clustering [45] where one representative conformation is taken from each cluster. The selected reduced decoys are then refined by energy minimization with the OPLS-AA force field [61] using GROMACS 4.0 simulation package [62] for the purpose of removing the steric clashes and regulating secondary structure details. However, the topology of the I-TASSER decoys is not changed by the energy minimization. A full set of I-TASSER decoys is downloadable at http://zhanglab.ccmb.med.umich.edu/decoys.

### Availability of RW potentials

RW and RWplus can be automatically derived by the CalRW program, which is freely downloadable at http://zhanglab.ccmb.med.umich.edu/RW. This section should provide enough detail to allow full replication of the study by suitably skilled investigators. Protocols for new methods should be included, but well-established protocols may simply be referenced. We encourage authors to submit, as separate supporting information files, detailed protocols for newer or less well-established methods. These are published online only, but are linked to the article and are fully searchable.
